# What are patient factors associated with the quality of diabetes care?: results from the Korean National Health and Nutrition Examination Survey

**DOI:** 10.1186/1471-2458-12-689

**Published:** 2012-08-22

**Authors:** Ki Dong Ko, Bo Hyun Kim, Sang Min Park, Soo In Oh, Chun Sik Um, Dong Wook Shin, Hae Won Lee

**Affiliations:** 1 Department of Family Medicine, Seoul National University Hospital, Seoul National University College of Medicine, Seoul, 110-744, Republic of Korea

**Keywords:** Diabetes mellitus, Quality indicators, Process assessment, Outcome assessment

## Abstract

**Background:**

Recently there has been a growing interest in healthcare quality control in Korea. We examined the association between patient factors and quality indicators of diabetic care among Korean adults with diabetes.

**Methods:**

We obtained a sample of 335 adults aged 20 or older diagnosed with diabetes from the 2005 Korean National Health and Nutrition Examination Survey. Patient factors were divided into two categories: socioeconomic position and health-related factors. Quality indicators for diabetes care were defined as receiving preventive care services for diabetes complications (e.g., fundus examination, microalbuminuria examination, diabetes education) and diabetes-related clinical outcomes (e.g., HbA1c, blood pressure, LDL-cholesterol). We performed multiple logistic regression analyses for each quality indicator.

**Results:**

We found that people with lower education levels or shorter duration of diabetes illness were less likely to receive preventive care services for diabetes complications. Women or people with longer duration of diabetes were less likely to reach the glycemic target. Obese diabetic patients were less likely to accomplish adequate control of blood pressure and LDL-cholesterol.

**Conclusions:**

Several factors of patients with diabetes, such as education level, duration of illness, gender, and obesity grade are associated with the quality of diabetes care. These findings can help inform policy makers about subpopulations at risk in developing a public health strategy in the future.

## Background

Diabetes has become a major health threat to Koreans. The prevalence of diabetes was estimated to be 9.1% (1.42 million people,10.2% of men and 1.17 million people, 7.9% of women) of Korean adults aged 30 years and over according to an analysis of the third Korean National Health and Nutrition Examination Survey, 2005 (KNHANES III) [1]. Diabetes was also rated as the fourth leading cause of death in 2005. The mortality rate due to diabetes was 24.5 per 100,000 persons [2]. Major causes of diabetes-related morbidity and mortality are macrovascular and microvascular complications. Diabetes is the leading cause of new cases of blindness in adults and gives rise to non-traumatic lower extremity amputations, end-stage renal diseases, and cardiovascular diseases [3]. Therefore, it is essential that appropriate management of patients with diabetes include early detection and prevention of complications.

Quality indicators assessing diabetes care have been widely developed and applied as a public health strategy in many countries [4]. For instance, the National Diabetes Quality Improvement Alliance (NDQIA) that involves 13 American organizations, including the American Diabetes Association (ADA), chose and set quality indicators: process measures (annually receiving one or more Hb A1c tests/at least one lipid profile/any test for microalbuminuria/dilated retinal eye examination/foot examination/influenza immunization, aspirin use, smoking cessation, pregnancy counseling) and outcome measures (HbA1c, LDL-cholesterol, microalbuminuria, blood pressure [BP]) [5]. These quality indicators were selected based on evidences demonstrating that adherence to those standards improved diabetic care and patient outcomes [6-11].

One of the major weak points of the South Korean health care system is the lack of attention and effort to policies related to the quality of care [12]. Recently there has been increasing interest in healthcare quality control in Korea. Therefore in areas of diabetes, quality indicators were recommended by the Korean Diabetes Association (KDA), which are similar to those of NDQIA [13].

There are numerous studies assessing the achievement of quality indicators for diabetes care in various healthcare settings [1,14-17]. However, little is known about the characteristics of diabetes patients associated with the quality indicators. Information on patient factors associated with the quality of diabetes care can be helpful to improve the quality of diabetic care. Therefore, we aimed to examine patient factors associated with preventive care services for diabetic complications and diabetes-related clinical outcomes in Korean adults with diabetes. To our knowledge, this is the first study to determine patient factors related to quality of diabetes care in Korea.

## Methods

### Subjects

The KNHANES III was approved by the Korean Ministry of Health and Welfare and conducted in 2005, in accordance with the ethical principles for medical research involving human subjects as defined by the Helsinki Declaration. It was a nationwide representative survey using a stratified, multistage probability sampling design for the selection of household units. The study participants provided written informed consent.

This survey consisted of three components: the Health Interview Survey, the Health Examination Survey, and the Nutrition Survey. The Health Interview Survey and the Nutrition Survey were conducted using self-administered questionnaires. Trained nurses took the lead in the Health Examination Survey, performing anthropometric measurements, BP measurement, and blood serum collection.

5463 people aged 20 years and over participated in the Health Interview Survey and the Health Examination Survey. Of these, 327 patients that had doctor-diagnosed diabetes were included in the study sample, excluding 12 subjects with incomplete responses to patient factors.

### Patient factors

The Health Interview Survey was used to gather information about socioeconomic factors (e.g., age, gender, education level, and household monthly income) and health-related factors (e.g., duration of diabetes illness, self-rated health, regular exercise). The age variable was stratified into three groups (20-49, 50-64, ≥65). Education level was classified into three groups (elementary school or lower, middle or high school, college or higher). The household monthly income variable was categorized into three groups (≤ 1000000 South Korean won [KRW], 1500000 KRW < income ≤ 3000000 KRW, > 3000000 KRW). The duration of diabetes was defined as the number of years since the patient received the diagnosis of diabetes by a doctor, and the variable was divided into three groups (<5 years, 5-9 years, ≥10 years). Self-rated health (SRH) status was assessed using the following question: “What is your current subjective health status?” Responses were categorized into three groups (excellent or good, fair, poor or very poor). The question, “Do you practice regular exercise in leisure time?” was used to classify the study sample into dichotomous groups according to the presence of regular exercise.

The Health Examination Survey was used to collect information about anthropometric data (i.e. height, body weight). BMI was calculated by dividing the weight in kilograms by the square of the height in meters and classified into three categories according to the cut-off criteria of WHO Western Pacific Region (BMI < 23, normal weight; 23 ≤ BMI < 25, overweight; BMI ≥ 25, obesity) [18].

### Quality indicators of diabetes care

We used the treatment guideline of the KDA to define the quality indicators for diabetes care. In this study, quality indicators for diabetes care were divided into two dimensions: preventive care services for diabetes complications and diabetes-related clinical outcomes (Table 1). The Health Interview Survey and the Health Examination Survey were used to obtain information about preventive care services for diabetes complications and diabetes-related clinical outcomes, respectively.

**Table 1:** Quality indicators for diabetes care in this study

**Quality indicators**	**Definition**
Preventive care services for diabetes complication	
Fundus exam	Received fundus examination in the past year
Microalbuminuria exam	Received microalbuminuria examination in the past year
Diabetes education	Received diabetes education ever
Diabetes-related clinical outcomes	
HbA1c	Level of HbA1c below 6.5%
Blood pressure (BP)	Level of systolic/diastolic BP below 130/80 mmHg
LDL-cholesterol	Level of LDL-cholesterol below 100 mg/dl

Preventive care services for diabetes complications consisted of fundus examination, micro albuminuria examination, and diabetes education. The questions, “Have you ever received the examination within the past year?” and “Have you ever received education about diabetes in the hospital or public health center?” were used to determine whether the patients had received appropriate preventive services for diabetes complications. Diabetes-related clinical outcomes consisted of HbA1c, BP, and LDL-cholesterol. Each cut-off level of each diabetes-related clinical outcomes was HbA1c < 6.5%, systolic BP < 130 mmHg and diastolic BP < 80 mmHg, LDL-cholesterol < 100 mg/dl according to the KDA guideline [13].

### Statistical methods

For statistical analyses, non-conditional multiple logistic regression was performed to determine which patient factors were associated with quality indicators. Patients with missing values in quality indicator variables were excluded from the analyses. Adjusted odds ratios (AORs) with 95% confidence intervals (CIs) were calculated after adjusting for age, gender, education level, household monthly income, duration of diabetes, self-rated health, regular exercise, and BMI. All estimates were weighted to represent the Korean adults with diabetes. Statistical analyses were performed using STATA 10.0 for Windows (StataCorp, LP, and College Station, Texas, USA).

## Results

### Characteristics of the study sample

Characteristics of the study sample (n = 327) aged 20 years or older who had doctor-diagnosed diabetes are presented in Table 2. Over 80% of subjects were ≥ 50 years of age. The study sample consisted of 51.1% men and 48.9% women. The largest proportion of the study sample were comprised of subjects with ‘elementary school or lower’ level of education and the lowest tertile of household income (49.5% and 44.7%, respectively). In the study sample, 47.1% had ‘less than 5 years’ duration of diabetes illness and 32.4% had ‘more than 10 years’ duration of diabetes illness. Over 60% of the study sample had very poor or poor SRH. 52.9% of the study sample had no regular exercise, and 41.9% were obese according to the WHO Western Pacific Region criteria. The quality of preventive care services for diabetes complications in Korea was not optimal (Table 3). Approximately 34% received a dilated eye examination in the past year, 40% received a microalbuminuria test in the past year, and 29% had received diabetes education. The quality status of diabetes-related clinical outcomes showed similar results. About 25% and 37% of patients with diabetes achieved HbA1c < 6.5% and BP < 130/80 mmHg, respectively. Only 28% reported LDL-cholesterol < 100 mg/dl.

**Table 2:** Characteristics of the study sample

**Variables**	**Diabetes mellitus patients (n = 327)**
Socioeconomic position	
Age (years)	
20-49	54 (19.7)
50-64	149 (44.4)
≥65	124 (35.9)
Gender	
Men	167 (52.0)
Women	160 (48.0)
Education level	
Elementary school or lower	162 (46.3)
Middle or high school	133 (43.0)
College or higher	32 (10.7)
Household income (monthly, thousand KRW)	
>3,000	49 (16.5)
>1,000 & ≤3,000	132 (41.7)
≤1,000	146 (41.9)
Health-related factors	
Duration of diabetes illness	
<5 years	154 (47.1)
5-9 years	67 (20.8)
≥10 years	106 (32.1)
Self-rated health	
Poor/very poor	200 (59.0)
Fair	90 (28.5)
Excellent/good	37 (12.5)
Regular exercise	
Yes	154 (47.6)
No	173 (52.4)
Body mass index	
<23	92 (27.5)
≥23 & <25	98 (29.6)
≥25	137 (43.0)

Data are n (%) unless otherwise indicated.

Percents are weighted to represent the Korean population with diabetes, aged 20 years or older.

**Table 3:** Factors associated with preventive care processes for diabetes complication in this study

**Variables**	**Fundus examination (n = 326)**			**Microalbuminuria examination(n = 327)**			**Diabetes mellitus education (n = 323)**		
**%**^**1**^	**%**^**2**^	**34.4**		**%**^**2**^	**40.0**		**%**^**2**^	**30.2**	
		**AOR**^**3**^	**95% CI**		**AOR**^**3**^	**95% CI**		**AOR**^**3**^	**95% CI**
Socioeconomic position									
Age (years)									
20-49	35.0	1.00		27.5	1.00		38.9	1.00	
50-64	40.6	1.40	0.69-2.84	45.7	3.07	1.38-6.84	31.0	0.70	0.33-1.52
≥65	26.8	0.65	0.26-1.64	39.5	2.74	1.10-6.80	24.8	0.51	0.20-1.31
Gender									
Men	31.5	1.00		38.9	1.00		31.2	1.00	
Women	37.8	1.88	0.98-3.61	41.3	0.95	0.52-1.74	29.0	1.37	0.69-2.70
Education									
Elementary or lower	31.0	1.00		39.0	1.00		23.7	1.00	
Middle or high school	30.8	1.25	0.61-2.59	36.8	1.11	0.59-2.08	32.6	1.89	0.91-3.92
C College or higher	63.0	6.61	2.27-19.24	56.7	3.91	1.35-11.26	47.6	4.75	1.52-14.84
Household income (monthly, thousand KRW)									
>3000	46.6	1.00		53.0	1.00		32.7	1.00	
>1000 & ≤3000	0 30.6	0.64	0.26-1.56	34.3	0.47	0.25-0.90	27.7	0.87	0.43-1.79
≤1000	33.2	0.92	0.39-2.14	40.3	0.59	0.27-1.26	31.5	1.51	0.67-3.39
Health-related factors									
Duration of diabetes illness									
<5 years	19.7	1.00		31.3	1.00		21.3	1.00	
5-9 years	39.8	2.42	1.11-5.28	38.5	1.40	0.70-2.82	25.3	1.13	0.49-2.59
≥10 years	52.3	6.66	3.33-13.31	53.9	2.96	1.50-5.83	46.9	4.47	2.23-8.93
Self-rated health									
Poor/very poor	38.6	1.00		44.0	1.00		30.9	1.00	
Fair	29.4	0.54	0.28-1.04	40.0	0.80	0.43-1.47	30.4	0.81	0.37-1.76
Excellent/good	26.8	0.39	0.13-1.12	23.0	0.32	0.12-0.86	26.8	0.82	0.30-2.24
Regular exercise									
Yes	39.5	1.00		41.1	1.00		31.6	1.00	
No	29.8	0.70	0.39-1.24	39.0	0.97	0.57-1.65	28.9	0.91	0.49-1.71
Body mass index									
<23	42.5	1.00		38.2	1.00		35.1	1.00	
≥23 & <25	28.8	0.42	0.17-1.01	37.2	0.79	0.40-1.59	29.7	0.79	0.31-2.04
≥25	31.0	0.56	0.29-1.09	42.2	1.16	0.62-2.19	24.3	0.64	0.33-1.23

Abbreviations: *AOR* adjusted odds ratio; *CI* confidence interval.

Weighted to represent the Korean population with diabetes, aged 20 years or older.

^1^ Proportion of subjects who tested fundus/microalbuminuria examination in the past year and ever received diabetes mellitus education.

^2^ Proportion of subjects who tested fundus/microalbuminuria examination in the past year and ever received diabetes mellitus education in each category of each variable.

^3^ AORs were obtained after adjusting for age, gender, education level, household monthly income, duration of diabetes, self-reported health status, regular exercise, and body mass index.

### Factors associated with preventive care services for diabetes complications

Results from regression analyses showed the association between patient factors and preventive care services for diabetes complications after controlling for other covariates (Table 3). Compared with the reference group of patients with ‘elementary or lower’ level of education, people with ‘college or higher’ level of education were more likely to receive preventive care services for diabetes complications (for fundus examination, AOR 6.61, 95% CI 2.27-19.24; for microalbuminuria examination, AOR 3.91, 95% CI 1.35-11.26; for diabetes education, AOR 4.75, 95% CI 1.52-14.84). Compared with the reference group of patients with < 5 years’ duration of diabetes illness, people whose duration was more than 10 years were more likely to receive preventive care for diabetes complications (for fundus examination, AOR 6.66, 95% CI 3.33-1331; for microalbuminuria examination, AOR 2.96, 95% CI 1.50-5.83; for diabetes education, AOR 4.47, 95% CI 2.23-8.93). The estimated AORs showed consistent and positive gradients by education level and duration category of diabetes illness.

### Factors associated with diabetes-related clinical outcomes

Table 4 presents the association between patient factors and diabetes-related clinical outcomes. The duration of diabetes illness affected glycemic control (HbA1c < 6.5%). Compared with the reference group of patients with < 5 years’ duration of diabetes illness, diabetes patients who were afflicted with the disease for more than 5 years were less likely to reach the glycemic target (AOR 0.32, 95% CI 0.11-0.95 in people with duration of 5-9 years; AOR 0.26, 95% CI 0.11-0.66 in people with ≥ 10 years’ duration). Women were less likely to reach the glycemic target (AOR 0.34, 95% CI 0.12-0.96). Compared with BMI < 23, diabetes patients with obesity also appeared to have lower compliance with the recommended targets of BP and LDL-cholesterol (for BP < 130/80 mmHg, AOR 0.43, 95% CI 0.23-0.81; for LDL-cholesterol < 100 mg/dl, AOR 0.28, 95% CI 0.12-0.66).

**Table 4:** Factors associated with diabetes-related clinical outcomes in this study

**Variables**	**HbA1c < 6.5% (n = 239)**			**BP <130/80 mmHg (n = 327)**			**LDL-C <100 mg/dl (n = 287)**		
**%**^**1**^	**%**^**2**^	**23.6**		**%**^**2**^	**37.1**		**%**^**2**^	**26.6**	
		**AOR**^**3**^	**95% CI**		**AOR**^**3**^	**95% CI**		**AOR**^**3**^	**95% CI**
Socioeconomic position									
Age (years)									
20-49	10.4	1.00		50.1	1.00		34.3	1.00	
50-64	28.5	5.61	1.03-30.57	33.1	0.56	0.25-1.23	21.4	0.52	0.20-1.36
≥65	22.7	4.90	0.88-27.30	34.8	0.47	0.20-1.13	28.5	0.53	0.19-1.51
Gender									
Men	27.9	1.00		34.2	1.00		27.8	1.00	
Women	19.4	0.34	0.12-0.96	40.3	1.35	0.75-2.43	25.3	0.74	0.36-1.52
Education									
Elementary or lower	24.2	1.00		37.6	1.00		29.4	1.00	
Middle or high school	28.7	1.06	0.45-2.45	32.4	0.70	0.38-1.28	21.4	0.48	0.23-1.02
College or higher	12.0	0.37	0.07-2.07	53.2	1.79	0.65-4.89	33.0	0.85	0.26-2.71
Household income (monthly, thousand KRW)									
>3,000	19.2	1.00		41.3	1.00		13.5	1.00	
>1,000 & ≤3,000	28.0	0.84	0.23-3.07	40.0	0.93	0.40-2.16	32.3	2.69	0.81-8.94
≤1,000	21.1	0.45	0.11-1.75	32.1	0.74	0.30-1.83	25.8	2.17	0.62-7.59
Health-related factors									
Duration of diabetes illness									
<5 years	35.1	1.00		41.4	1.00		24.6	1.00	
5-9 years	16.5	0.32	0.11-0.95	35.4	0.71	0.36-1.41	27.2	1.10	0.45-2.69
≧10 years	16.0	0.26	0.11-0.66	31.3	0.61	0.32-1.15	28.2	1.07	0.51-2.23
Self-rated health									
Poor/very poor	26.2	1.00		37.4	1.00		28.6	1.00	
Fair	16.7	0.41	0.15-1.15	39.1	0.98	0.50-1.90	19.2	0.62	0.28-1.37
Excellent/good	33.6	1.52	0.50-4.62	30.3	0.61	0.25-1.46	32.2	1.17	0.42-3.26
Regular exercise									
Yes	21.0	1.00		36.8	1.00		24.8	1.00	
No	27.3	1.56	0.73-3.32	38.0	1.07	0.59-1.93	27.6	0.98	0.50-1.94
Body mass index									
<23	21.1	1.00		48.1	1.00		43.8	1.00	
≥23 & <25	20.5	1.21	0.44-3.37	38.6	0.73	0.38-1.39	24.2	0.45	0.20-0.99
≥25	26.9	1.41	0.63-3.16	29.3	0.43	0.23-0.81	17.7	0.28	0.12-0.66

Abbreviations: *BP* blood pressure; *LDL-C* LDL-cholesterol; *AOR* adjusted odds ratio; *CI* confidence interval.

Weighted to represent the Korean population with diabetes, aged 20 years or older.

^1^ Proportion of subjects who reached HbA1c < 6.5%, BP <130/80 mmHg, LDL-C <100 mg/d.

^2^ Proportion of subjects who reached HbA1c < 6.5%, BP <130/80 mmHg, LDL-C <100 mg/d in each category of each variable.

^3^ AORs were obtained after adjusting for age, gender, education level, household monthly income, duration of diabetes, self-reported health status, regular exercise, and body mass index.

## Discussion

This study examined patient factors associated with quality indicators for diabetes care in South Korea. Our study is unique in that it included the quality indicators of preventive care processes for diabetic complications and diabetes-related clinical outcomes comprehensively. In this study, we found that those with lower education level and shorter duration of diabetes illness had relatively less experience in receiving preventive care services for diabetes complications. Patients with longer duration of diabetes illness and women showed poor glycemic control. Additionally, obese diabetic patients were less likely to accomplish adequate control of blood pressure and LDL-cholesterol.

Our analyses resulted in 5 key findings. Firstly, our findings showed that people with lower education level were less likely to have received fundus and microalbuminuria examinations in the past year, and education about diabetes as preventive care services for diabetes complications. Educational level, the most widely used measure of socioeconomic position, imparts health-related knowledge capacity, reflects access to resources including preventive health care, and determines health behaviors [19,20], which may explain our results. We can assume that those with a lower level of education have inadequate understanding about possible diabetic complications and fewer opportunities to meet physicians, and thus are less likely to receive appropriate preventive care services for diabetes complications. Previous studies have shown that diabetes patients with lower levels of education have lower rates of eye examinations [21-23]. A study in 2011 demonstrated that lower education level was associated with poor achievement of services such as dilated eye examination, microalbuminuria test, and diabetes education [23]. These results can imply that stronger public health efforts are needed to increase rates of receiving preventive care for diabetes complications among those with lower education levels. On the other hand, education level was not associated with diabetes-related clinical outcomes in our study. Additionally, Haffner SM et al. found no association between education level and glycemic control in Mexican Americans [24]. Our results revealed the discordance in association of education level with preventive care services for diabetes complications and diabetes-related clinical outcomes. After controlling for other covariates, education level could be an independent factor associated with receiving preventive care services for diabetes complication, but not with clinical outcomes.

Secondly, income level did not seem to affect quality indicators in this study. Several studies found that lower income was related to poor quality of diabetic care [23,25,26]. A variety of reasons explaining this association have been suggested, such as medical costs of preventive care and medication as well as psychosocial factors [23]. In our study, however, there were hardly any differences in the quality indicators based on income level, which could be attributable to the use of a universal health insurance system in South Korea. The National Health Insurance covers the majority of citizens (96%), while the Medical Aid program covers the poor and other specified groups (4%) [27]. Since the financial burden is low within the healthcare system, individual income level is less likely to play an important role [28].

Thirdly, in our study, shorter duration of diabetes illness was associated with lower levels of receiving preventive care services for diabetes complications and longer duration was associated with lower achievement of recommended glycemic goal. In the cross-sectional analysis of Indian Health Service Diabetes Care and Outcomes Audit, < 5 years’ duration of diabetes illness was strongly associated with poor reception of preventive care services for diabetes complications [29]. Recently-diagnosed diabetes patients may have lower reception of preventive care for diabetes complications because they have not received as much education about diabetes complications and recommendation to get preventive care from physicians than patients with longer diabetes duration. Additionally, patients who have been recently diagnosed might have fewer complications, and thus are not as motivated to receive preventive diabetes care [30]. preventive care services for diabetes complications According to the UK Prospective Diabetes Study (UKPDS), the proportion of patients who achieved the target HbA1c level below 7% HbA1c was only 50% of the patients and this percentage decreased dramatically as the duration of illness increases even with intensive treatment [6]. The major reason for poor glycemic control in people with long duration of diabetes may be due to disease progression following the natural course of diabetes.

Fourthly, women were less likely to reach the glycemic target level. Up to date, there is a lack of consistency in the findings about gender differences in glycemic control among patients with diabetes [31-34]. A few studies have indicated that female patients are at greater risk of not achieving the recommended HbA1c levels [31,32], while other studies found no significant gender-related differences [33,34]. There may be several possible explanations for lower achievement rates of glycemic target levels in female diabetes patients, such as fewer opportunities for treatment, less aggressive treatment, sex-based physiology, in which therapeutic interventions such as diet or drug therapy may not be as effective [31]. Gender differences in our study were detected only for glycemic control. In contrast, in a cross-sectional study of 5082 men and 4293 women in Sweden, female patients had poor control for all clinical outcomes (HbA1c, BP, and LDL-cholesterol) than corresponding male patients in the subgroup aged 60-75 [32]. Further studies with larger data using age-group analyses are necessary for investigating gender differences in clinical outcomes management in Korea.

Finally, obese patients with diabetes were less likely to meet the target values of BP and LDL-cholesterol, although we failed to prove an association between specific obesity grades and glycemic control. In one study, Harris et al. revealed that BMI was not related to glycemic control, which is consistent with our result [35]. One possible explanation for this is that BMI could both affect glycemic control and be influenced by the level of glycemic control. For example, a low BMI causes insulin sensitivity and therefore good glycemic control. Improved glycemic control causes weight gain, which is a finding consistent with the UKPDS, where the intensive group gained 2 to 5 kg compared with the conventional group [6]. As weight increases, glycemic control worsens over time; thus BMI and glycemic control are coupled with bi-directional influence. Furthermore, overall obesity as determined by BMI values in our study could be less strongly associated with insulin resistance and poor glycemic control than abdominal obesity [36]. Similar to our results, previous studies have also showed a positive association between BMI and BP or LDL-cholesterol [37,38]. This provides a meaningful lesson for physicians to pay attention to BP and LDL-cholesterol levels among obese diabetes patients.

There are some limitations in our study. Firstly, since our study was based on a cross-sectional design, there was no information on the temporal relationship and therefore, causal associations were unable to be made. Thus, prospective studies are helpful to determine the causal effect of patient factors on quality indicators. Secondly, we could not consider all possible patient factors which may include confounding factors, and account for other important outcome measures (e.g., foot examination, anti-platelet therapy, and smoking cessation etc.) due to lack of information from the KNHANES III. Thirdly, our study used self-administered questionnaires for a majority of the information, which may be subject to recall bias. For instance, patients might not know that they had been tested for microalbuminuria. There were also relatively many missing values in the HbA1c and LDL-C variables. The excluded and included patients for the analyses may have different associations between patient factors and quality indicators, which can lead to non-response bias. Lastly, since non-biometric categorical variables may have low reliability and may not capture complex characteristics of interest very well, differences that exist are masked. For example, in our study, exercise was roughly divided into two groups according to regular exercise in leisure time. Quantitative classification of exercise using metabolic equivalent of task (MET) may be a better choice to grasp the differences in detail.

## Conclusions

Achievement of quality indicators in diabetic patients is well known to help accomplish the goals of public health: living longer and feeling better [6-11]. Several factors of patients with diabetes, such as education level, duration of illness, gender, and obesity grade were associated with the quality indicators of diabetes care in this study. Our findings can help inform policy makers about subpopulations at risk in developing a public health strategy in the future.

## Competing interests

The authors declare that they have no competing interests.

## Authors’ contributions

SP was involved in the conception and design of the study. SO, CU, and DS contributed to data analysis and the interpretation of data. KK and BK drafted the manuscript, participating in all areas of the study. HL participated in improving the quality of written English and critically revising the manuscript. All authors read and approved the final manuscript.

## Pre-publication history

The pre-publication history for this paper can be accessed here:

http://www.biomedcentral.com/1471-2458/12/689/prepub
